# Interstitial pneumonias of undetermined etiology in foals in California, 1990–2020

**DOI:** 10.1177/10406387251410524

**Published:** 2026-01-29

**Authors:** Javier Asin, Francisco Carvallo, Omar A. Gonzales-Viera, Melissa Macías-Rioseco, Nicolas Streitenberger, Sahar Abdelrazek, Beate Crossley, Patricia A. Pesavento, Francisco A. Uzal

**Affiliations:** San Bernardino laboratories, California Animal Health and Food Safety Laboratory System, University of California–Davis, Davis, CA, USA; Department of Pathology, Microbiology, and Immunology, University of California–Davis, Davis, CA, USA; Virginia-Maryland College of Veterinary Medicine, Virginia Polytechnic Institute and State University, Blacksburg, VA, USA; Davis laboratories, California Animal Health and Food Safety Laboratory System, University of California–Davis, Davis, CA, USA; Department of Pathology, Microbiology, and Immunology, University of California–Davis, Davis, CA, USA; Tulare laboratories, California Animal Health and Food Safety Laboratory System, University of California–Davis, Davis, CA, USA; Davis laboratories, California Animal Health and Food Safety Laboratory System, University of California–Davis, Davis, CA, USA; Department of Pathology, Microbiology, and Immunology, University of California–Davis, Davis, CA, USA; Virginia-Maryland College of Veterinary Medicine, Virginia Polytechnic Institute and State University, Blacksburg, VA, USA; Davis laboratories, California Animal Health and Food Safety Laboratory System, University of California–Davis, Davis, CA, USA; Department of Pathology, Microbiology, and Immunology, University of California–Davis, Davis, CA, USA; San Bernardino laboratories, California Animal Health and Food Safety Laboratory System, University of California–Davis, Davis, CA, USA; Department of Pathology, Microbiology, and Immunology, University of California–Davis, Davis, CA, USA

**Keywords:** acute lung injury, acute respiratory distress syndrome, foals, interstitial pneumonia

## Abstract

Interstitial and bronchointerstitial pneumonias of undetermined etiology in young foals are relatively common in autopsy services with an equine focus. Unknown viruses, toxins, hyperthermia, surfactant or alveolar macrophage function deficiency, certain antibiotics, and aberrant responses to *Rhodococcus equi* or other bacteria have been proposed as causes. We performed a retrospective study of autopsies on foals with a diagnosis of interstitial or bronchointerstitial pneumonia with an unidentified etiology. Forty-one foals (median age: 3-mo-old) were included. Most were received in summer (*n* = 28) and spring (*n* = 10). The most frequently reported clinical signs were dyspnea and/or tachypnea (*n* = 28) and fever (*n* = 19). Antibiotic treatment was reported in 21 cases, and the most frequently used antibiotics were penicillin (*n* = 9) and gentamicin (*n* = 8). Grossly, most of the lungs were diffusely rubbery-to-firm (*n* = 35) and did not collapse (*n* = 22). Histologically, combinations of exudative (E; hyaline membranes), proliferative (P; type II pneumocyte hyperplasia), and fibrotic (F; fibroplasia) phases were common (E + P, *n* = 15; E + P + F, *n* = 13) in the interstitial component. Necrosis of the bronchiolar epithelium was rare (*n* = 4), concurrent suppurative bronchopneumonia was common (*n* = 22), and a few foals (*n* = 5) had pulmonary pyogranulomas. *Pneumocystis* spp. organisms were observed in 8 cases using Grocott–Gomori methenamine silver stain. Bacteria were recovered from the lungs in 22 cases, with *R. equi* (*n* = 7) and *E. coli* (*n* = 6) being the most common isolates. No unequivocal viral causes were identified during the regular diagnostic work-up and after using novel diagnostic approaches such as herpesvirus consensus PCR and viral metagenomics in a subset of the cases.

Interstitial and bronchointerstitial pneumonias of foals with features similar to acute lung injury (**ALI**) and/or acute respiratory distress syndrome (**ARDS**) have posed significant diagnostic challenges over the years.^4,10,26^ There are scattered reports from North America and Europe since the late 1980s,^8,25^ and the current consensus is that these cases represent a syndrome with several contributing factors rather than a specific disease with a single etiology.^13,26^

The age of the affected foals varies slightly across the reports, but most studies include animals of 1–8-mo-old.^13,15,26,28^ These cases usually occur in clusters within groups (e.g., in breeding farms, with multiple cases occurring within 6 of 10 farms in a report^15^). Affected foals clinically have severe respiratory distress and hyperthermia.^15,26^ At autopsy, lungs are non-collapsed and rubbery.^26^ Histologically, interstitial lesions range from acute hyaline membrane formation to subacute-to-chronic type II pneumocyte hyperplasia and fibrous tissue deposition.^15,26^ Inflammation and/or necrosis in the bronchi and/or bronchioles are described in some cases.^10,15,26^ Occasionally, there is concomitant bacterial bronchopneumonia and/or pyogranulomas caused by *Rhodococcus equi*.^4,10^ Some foals reportedly survive and recover pulmonary function.^26^

Initial reports suggested a toxic or a viral cause as the inciting factor.^8,25^ Interstitial pneumonias associated with exposure to plants such as perilla mint (*Perilla frutescens*) or Crofton weed (*Ageratina adenophora*) have been described in equids.^5,30^ Equid alphaherpesvirus 1 (EqAHV1; family *Orthoherpesviridae*, taxon species *Varicellovirus equidalpha1*), equid alphaherpesvirus 4 (EqAHV4; *Varicellovirus equidalpha4*), and equine influenza A virus (equine IAV; family *Orthomyxoviridae*, taxon species *Alphainfluenzavirus influenzae*) have been sporadically detected in foals with interstitial or bronchointerstitial pneumonia^21,23,26^; however, fatal respiratory infections with EqAHV1 and equine IAV tend to occur more often in <1-mo-old foals; severe pneumonia associated exclusively with EqAHV4 is rare.^6,10,22^ Equid gammaherpesvirus 2 (EqGHV2; family *Orthoherpesviridae*, taxon species *Percavirus equidgamma2*) has been detected in some cases of interstitial pneumonia in foals, and a putative role has been proposed.^3,26^ EqGHV2 is a ubiquitous agent in equine populations and can be detected by PCR in foals as young as 25-d-old^17,19^; however, viral products (i.e., antigen or nucleic acids) have never been visualized within lesions of interstitial pneumonia.

Two novel parvoviruses and a picornavirus were discovered in samples of foals with interstitial pneumonia in California using viral metagenomics.^2^ The molecular frequency of those viruses was similar in healthy horses and horses with fever and respiratory disease; therefore, a contributory role of these viruses was considered unlikely.^27^

Other proposed etiologies include the use of certain antibiotics, in particular erythromycin; hyperthermia; high environmental temperatures; defects in surfactant production and/or alveolar macrophage function; and aberrant responses to *R. equi* or other bacterial infections.^10,15^ *Pneumocystis carinii* infection can cause similar lung lesions in weaned foals.^1,10^

Our main objective in this retrospective study was to describe autopsy cases of foals with interstitial or bronchointerstitial pneumonia of undetermined etiology, using a large cohort of cases to establish whether there are distinctions in signalment, clinical signs, lesions, and co-pathogens. A secondary objective was to investigate other potential viral causes through deep sequencing and metagenomics, herpesvirus consensus PCR, and in situ hybridization (ISH).

## Materials and methods

### Selection criteria and data recorded

We searched the California Animal Health and Food Safety Laboratory System (**CAHFS**; Davis, San Bernardino, and Tulare laboratories, CA, USA) database for autopsy cases of 1–12-mo-old foals with a diagnosis of interstitial or bronchointerstitial pneumonia of undetermined etiology, received between 1990 and 2020. Only cases with histologic slides of lung available for re-evaluation were included. Information on signalment, season, geographic location (i.e., county), clinical history, gross findings, and histologic lesions in organs other than the lungs was recorded. The final reports were screened for the results of ancillary tests, including bacteriology (aerobic culture, *Mycoplasma* spp. culture, *Salmonella* spp. RT-qPCR and/or enrichment culture in lung and extrapulmonary sites), virology (EqAHV1 qPCR, IHC, and/or FA; EqAHV4 IHC and/or qPCR; equine IAV RT-qPCR and/or FA; and viral isolation from lung), and toxicology (mineral screen, including determinations of liver concentrations of arsenic, cadmium, copper, iron, lead, manganese, mercury, molybdenum, selenium, and zinc; and liver vitamin E). These tests were requested by the coordinating pathologist in each case and were performed following CAHFS standard operating procedures.

### Lung histology

Available H&E-stained lung sections (1–14/case; median: 4) were re-evaluated to determine the presence or absence of interstitial inflammatory infiltrates (which were further classified based on the predominant cell type), type II pneumocyte hyperplasia, hyaline membranes, interstitial fibroplasia, interstitial edema, bronchiolar epithelial necrosis, bronchiolar epithelial hyperplasia, bronchus- or bronchiole-associated lymphoid tissue (**BALT**) hyperplasia, peribronchiolar-to-intramural mononuclear infiltrates, alveolar macrophage proliferation, alveolar edema, multinucleate cells, alveolar hemorrhage, suppurative bronchopneumonia, pyogranuloma(s), and bacteria.

Cases were categorized into 3 interstitial pneumonia phases, or combinations of exudative (with hyaline membranes), proliferative (with type II pneumocyte hyperplasia), and fibrotic (with interstitial fibroplasia). Cases with the absence of those 3 features and only interstitial inflammatory infiltrates were categorized as “non-classifiable”. At least one lung section from each case with available blocks of formalin-fixed, paraffin-embedded (FFPE) tissues was stained with Grocott–Gomori methenamine silver (GMS), and the presence or absence of *Pneumocystis* spp. forms was determined. Immunohistochemistry (IHC) was performed on lung sections from 3 selected cases with multinucleate cells using the following antibodies: pancytokeratin (LU5, mouse monoclonal, BioCare CM043C; 1:100), CD204 (SRA-E5, mouse monoclonal, TransGenic KT022; 1:200), and CD18 (2G1, mouse monoclonal, Peter Moore; 1:10).

### Herpesviral consensus PCR and ISH

Frozen lung and spleen samples from 5 cases from 2020 were subjected to herpesviral consensus PCR targeting a conserved region of the polymerase gene as described previously,^32^ followed by sequencing of selected obtained amplicons. These cases were selected because they were part of a recent cluster of interstitial pneumonias of undetermined etiology that we described previously.^2^ The same tissues from an EqAHV1-negative, 3-d-old foal with interstitial pneumonia of undetermined etiology (not included in our retrospective study) were also tested in parallel as a negative control because infection with other herpesviruses (i.e., gammaherpesviruses) is thought to occur slightly later in life.^17,19^ On FFPE lung tissue from the 5 cases, we performed colorimetric ISH for EqGHV2 (probe-V-EHV2-gB; cat. 463691) using RNAscope 2.5 HD reagent kit-RED (ACD) following the manufacturer’s instructions, and as described previously.^24^

### Deep sequencing and metagenomics

Deep sequencing and metagenomics (**Suppl. Table 1**) were performed on FFPE lung tissue from 3 cases (2 from 2020 [included in a previous study]^2^ and 1 from 2017; stored 4–7 y). We selected these 3 cases because they were recent and had better chances of containing viable genetic material. Tissues from 2 age-matched foals (1 from 2020 and 1 from 2018; stored 4–6 y) without interstitial pneumonia (not included in our retrospective study) were included in the runs as controls. Briefly, PromethION (4 of 5 cases) and MinION (1 of 5 cases) nanopore sequencing libraries were prepared following the protocols provided by Oxford Nanopore Technologies. Metagenomic reads were processed on Virginia Tech’s high-performance computing cluster (Advanced Research Computing, https://arc.vt.edu/). FASTQ files were used as an input for Kraken2 (v.2.1.2)^33^ to determine the taxonomic composition within the samples. Additionally, Minimap2 (v.2.24)^16^ was used to map reads in FASTQ format against equid gammaherpesvirus 5 (EqGHV5; *Percavirus equidgamma5*; GCF_000929435.1) and EqGHV2 (GCF_000843985.2) genomes from NCBI.

## Results

### Signalment and submission information

Forty-one foals fulfilled the inclusion criteria: 22 (54%) males, 17 (41%) females, and 2 (5%) with unreported sex. The median age was 3-mo-old (range: 1–12; interquartile range [IQR]: 2–4). Breed information was available in 39 of 41 cases. Thoroughbred and Quarter Horse foals were overrepresented (*n* = 23 [59%] and *n* = 13 [33%], respectively). Other breeds included Arabian, Appaloosa, and Welsh Pony (*n* = 1 [3%] each). An autopsy was performed in the laboratory in 37 cases, and there were 4 field autopsies performed by the submitting veterinarian. The foals were received in summer (*n* = 28 [68%]), spring (*n* = 10 [24%]), and fall (*n* = 3 [7%]); no cases were received in winter. The geographic origin was reported in 39 of 41 cases. Foals originated from the following California counties: Riverside (*n* = 18 [46%]); San Bernardino (*n* = 5 [13%]); San Joaquin (*n* = 4 [10%]); San Luis Obispo, Orange, San Diego, and Los Angeles (*n* = 2 [5%] each); and Sacramento, Placer, Sonoma, and Solano (*n* = 1 [3%] each).

### Clinical history

Clinical history was available in 40 of 41 cases. In 33 (82%) cases, the submitter mentioned that the foal had died spontaneously (i.e., not euthanized). There was no mention of the manner of death in 7 (18%) cases. In 30 cases, the submitter specified the duration of the clinical signs, with a median of 3.5 d (range: 0 [found dead with no prior signs of illness]–21; IQR: 1–7.5) reported. In 12 (30%) cases, there was a history of other foals with similar respiratory signs affected on the property. Of those, a specific number of affected foals was available in 2 cases (2 of 4, and 10 of 500 foals affected, respectively).

The most frequently reported clinical signs were dyspnea or tachypnea (*n* = 28 [70%]) and fever (*n* = 19 [48%]). Of 19 foals with body temperature data available, 14 had a fever with a median temperature of 40.3°C (104.6°F; range: 39.3–41.1°C [102.8–106.0°F]; IQR: 39.4–40.6°C [103.0–105.0°F]). Seven (18%) foals had a history of upper respiratory disease (e.g., nasal discharge, sneezing). Antibiotic treatment was reported in 21 (53%) cases. Of those, a specific antibiotic was reported in 19 cases, including penicillin (*n* = 9); gentamicin (*n* = 8); ceftiofur (*n* = 6); rifampicin (*n* = 5); erythromycin (*n* = 3); trimethoprim, sulfamethoxazole, and azithromycin (*n* = 2 each); and metronidazole, clarithromycin, amoxicillin, sulfamethoxazole, chloramphenicol, and doxycycline (*n* = 1 each). Combinations of 2 or more antibiotics were reported in 14 cases. Corticosteroid treatment was reported in 10 (25%) cases.

### Gross and histologic findings

**Figures 1–6.**
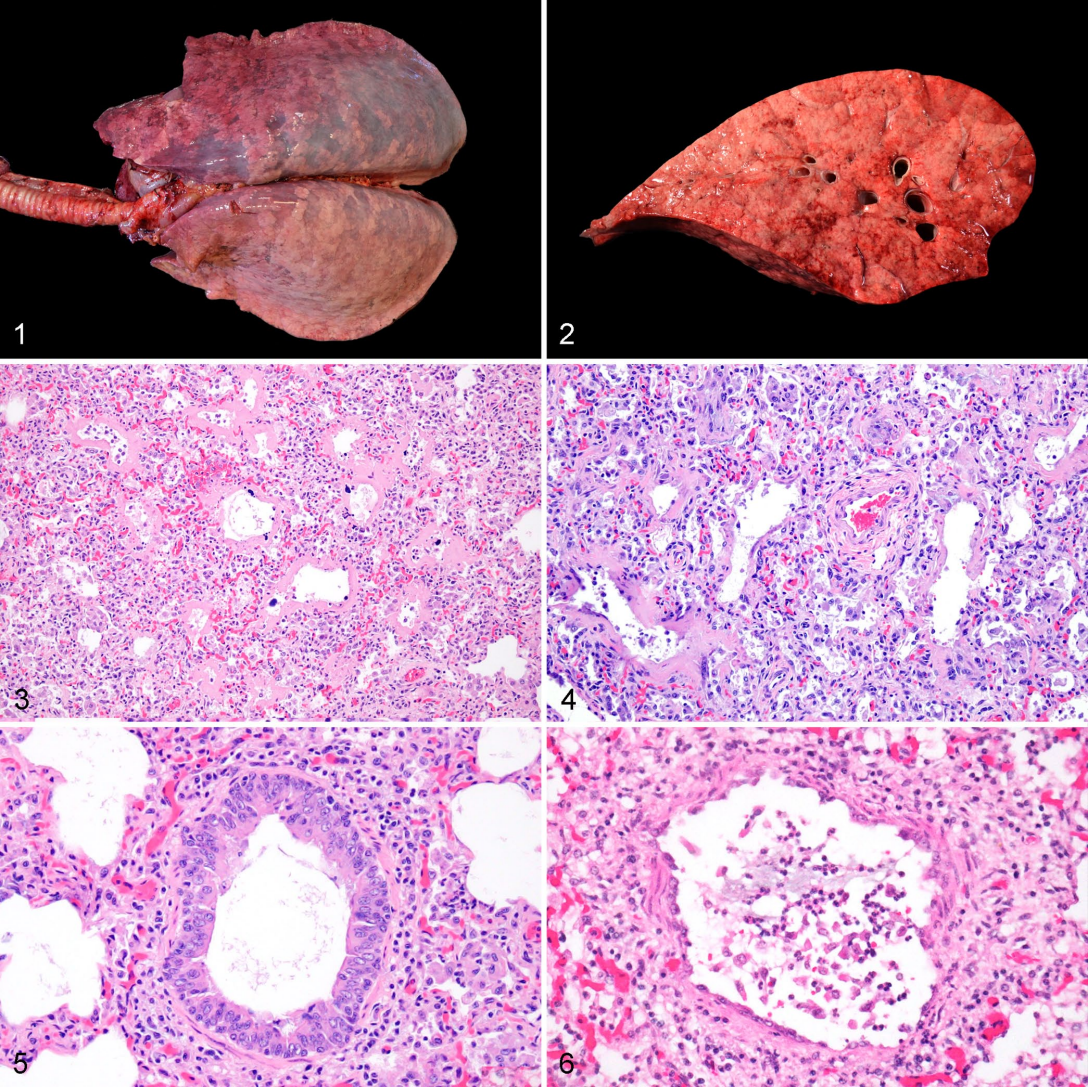
Gross and microscopic lesions in the lungs of foals with interstitial or bronchointerstitial pneumonia of undetermined cause. **Figures 1, 2.** Gross lesions. **Figure 1.** Lungs are diffusely swollen, non-collapsed, and have scattered lobular atelectasis. **Figure 2.** Cut section of a lung with smooth, bulging surface. **Figures 3–6.** Microscopic lesions. H&E. **Figures 3, 4.** Interstitial changes. **Figure 3.** Interstitial pneumonia, combination of exudative and proliferative phases. There are prominent hyaline membranes lining the alveoli and type II pneumocyte hyperplasia. **Figure 4.** Interstitial pneumonia, combination of exudative, proliferative, and fibrotic phases. There are hyaline membranes mixed with streams of fibroblasts that expand the interstitium, and type II pneumocyte hyperplasia. **Figures 5, 6.** Bronchiolar changes. **Figure 5.** Bronchiolar epithelial hyperplasia and peribronchiolar infiltrates of lymphocytes and plasma cells. **Figure 6.** Bronchiolar epithelial necrosis and peribronchiolar infiltrates of lymphocytes and plasma cells.

A complete gross description was available in 39 cases (**Table 1**). Nutritional condition, estimated by evaluating fat stores and musculature, was reported in 29 cases. Of those, 24 (83%) were in good, 1 (3%) was in fair-to-good, 3 (10%) were in fair, and 1 (3%) was in poor nutritional condition. Lungs were diffusely rubbery-to-firm (*n* = 35 [90%]), swollen and non-collapsed (*n* = 22 [56%]; **Figs. 1, 2**).

**Table 1.** Gross lesions in lungs and regional lymph nodes in 39 foals* with interstitial pneumonia.

Gross lesion	No. of foals (%)
Rubbery-to-firm texture	35 (90)
Non-collapsed	22 (56)
Lobular atelectasis	18 (46)
Thoracic lymphadenomegaly	17 (44)
Salmon-to-gray discoloration	13 (33)
Foam in airways	12 (30)
Pyogranulomas	4 (10)
Congestion	3 (8)
Consolidation	3 (8)
Hemorrhages	2 (5)

* In 2 of 41 foals, a macroscopic description of the lungs was not available.

Histologically (**Table 2**), all 41 cases had interstitial infiltrates of lymphocytes, plasma cells, and/or histiocytes. Combinations of different phases of interstitial pneumonia were common. Fifteen (37%) cases had a combination of exudative and proliferative phases (**Fig. 3**), and 13 (32%) cases were classified as combined exudative, proliferative, and fibrotic (**Fig. 4**). Five (12%) cases were categorized as purely exudative, 4 (10%) as a combination of proliferative and fibrotic, and 2 (5%) as purely proliferative. Two (5%) cases had only interstitial infiltrates and were left as non-classifiable. Of the cases with >1 lung section available and that were classifiable (37 of 41), 10 (27%) cases had different lesion age/phases across different sections, whereas in 27 (73%) cases, there was a mix of lesion age/phases in each section. In 23 of 41 (56%) cases, there were peribronchiolar aggregates of lymphocytes, plasma cells, and histiocytes, which occasionally infiltrated the wall (**Figs. 5, 6**). Bronchiolar epithelial necrosis was rare (*n* = 4 [10%]; Fig. 6), but epithelial hyperplasia was more frequent (*n* = 13 [32%]; **Fig. 5**). Twenty-two cases (54%) had aggregates of neutrophils in the lumen of the bronchioles and adjacent alveoli, consistent with suppurative bronchopneumonia, and 5 (12%) cases had pyogranulomas.

**Table 2.** Pulmonary histologic lesions in 41 foals with interstitial pneumonia.

Histologic lesion	No. of foals (%)
Interstitium	
Inflammatory infiltrates	41 (100)
Type II pneumocyte hyperplasia	34 (83)
Hyaline membranes	33 (80)
Interstitial edema	24 (58)
Fibroplasia	17 (42)
Airways	
Peribronchiolar-to-intramural mononuclear infiltrate	23 (56)
Bronchiolar epithelial hyperplasia	13 (32)
BALT hyperplasia	9 (22)
Bronchiolar epithelial necrosis	4 (10)
Alveoli	
Alveolar macrophage proliferation	40 (98)
Alveolar edema	30 (73)
Multinucleate cells	28 (68)
Alveolar hemorrhages	26 (63)
Other concomitant patterns	
Suppurative bronchopneumonia	22 (54)
Pyogranuloma(s)	5 (12)
Etiologic agents	
*Pneumocystis* sp. forms	8 (21)*
Bacteria	5 (12)

BALT = bronchus- or bronchiole-associated lymphoid tissue.

* Of 38 cases examined with Grocott–Gomori methenamine silver stain.

Multinucleate cells were observed in 28 (68%) cases, and they were present only in the alveolar lumen in all but 1 case, in which the multinucleate cells were also noted in the bronchiolar lumen. In 3 of 3 cases, the multinucleate cells were immunolabeled with anti-CD18 (membranous labeling) and anti-CD204 (cytoplasmic and membranous labeling), but not with anti-pancytokeratin antibodies and, therefore, the cells were interpreted as macrophagic in origin. *Pneumocystis* sp. forms were observed in 8 of 38 (21%) cases stained with GMS. In 7 of 8 cases, there were small numbers of organisms restricted to a few alveoli, whereas in 1 case, there were abundant, diffuse organisms. Bacteria were observed in 5 (12%) cases.

Information about extrapulmonary lesions was available in 39 cases. The main extrapulmonary, intra-thoracic lesions included lymphadenomegaly (*n* = 17 [44%]; which corresponded histologically to lymphoid hyperplasia) and pericardial effusion (*n* = 8 [20%]). One case (3%) had pyogranulomas in the mediastinal lymph nodes, and another case (3%) had pleural effusion. The most common extra-thoracic lesions (**Suppl. Table 2**) included lymphoid depletion (*n* = 10 [26%]), more frequently in the splenic white pulp, but also in the colonic mucosal-associated lymphoid tissue and lymph nodes, centrilobular hepatic degeneration and/or necrosis (*n* = 6 [15%]), and rare, random hepatocellular necrosis or suppurative hepatitis (*n* = 6 [15%]). Extrapulmonary, extra-thoracic lesions associated with *R. equi* included suppurative vertebral osteomyelitis and mesenteric lymphadenitis (*n* = 1 [3%] each). One case (3%) had tubulointerstitial, lymphoplasmacytic nephritis, and *Leptospira* spp. antigen was detected in the kidney by IHC.

### Bacteriology, virology, and toxicology results

Aerobic culture from the lung was performed in all 41 foals, and bacteria were recovered in 22 (54%) cases (**Suppl. Table 2**). The most common isolates were *R. equi* (*n* = 7 [17%]) and *Escherichia coli* (*n* = 6 [15%]). Other isolates included *Streptococcus equi* subsp. *zooepidemicus*, *Bordetella bronchiseptica*, and *Actinobacillus* sp. (*n* = 2 [5%] each). Mixed cultures were obtained in 15 of 22 cases. *Mycoplasma* spp. culture from the lung was negative in all 8 tested. *Salmonella* spp. were not detected in lung or extrapulmonary sites in any of the 32 cases tested.

Of the 22 cases that had suppurative bronchopneumonia histologically, bacteria were recovered in 12 (55%); in 7 of those cases, the recovered bacteria were interpreted as a very likely cause or contributor to the bronchopneumonia. Similarly, of the 5 cases with pyogranulomas, *R. equi* was recovered and considered the cause in 3 (60%).

Cultures in organs other than the lung were done in 35 cases. Of those, *R. equi* was isolated in extrapulmonary sites in 4 (11%) cases. The same bacteria were isolated from lung and at least one other parenchymal organ in 8 (23%) cases (**Suppl. Table 2**).

There was no detection of EqAHV1 (29 cases tested), EqHAV4 (12 cases tested), or equine IAV (17 cases tested). Virus isolation from lung was negative in all 17 cases tested.

Liver mineral screen was performed in 17 cases. Of those, 12 (70%) cases had lower than expected hepatic selenium concentration, with a median of 0.22 ppm (range: 0.10–0.29 ppm; IQR: 0.20–0.25 ppm; RI: 0.3–1.0 ppm). Liver vitamin E was measured in 3 of the cases with deficient hepatic selenium concentration, and it was below expected ranges in 1 case (1.7 ppm; RI: >3 ppm). The rest of the liver mineral concentrations were unremarkable.

### Herpesviral consensus PCR, ISH, and deep sequencing and metagenomics

Herpesviral consensus PCR yielded positive bands of similar size in both spleen and lung of 5 of 5 cases. In contrast, samples from the 3-d-old foal included in parallel in the run were negative. Sequencing of selected amplicons identified the herpesvirus as EqGHV2. No unequivocal EqGHV2 nucleic acid labeling was observed in the lung of any of the 5 cases tested by ISH. No significant reads were obtained in the deep sequencing and metagenomics analyses performed on the 3 cases and the 2 controls.

## Discussion

Cases of interstitial or bronchointerstitial pneumonia in young foals are common in autopsy services with an equine focus. A definitive cause for those lung lesions is usually not determined.^8,13,15,25,26^ Here we report 41 autopsy cases of 1–12-mo-old foals with interstitial or bronchointerstitial pneumonia with no definitive etiology identified that were received in 3 California veterinary diagnostic laboratories over a period of 30 y. These criteria of inclusion were established based on previous descriptions of this condition. The age range, in particular, was chosen to ensure that all of the ranges reported in the literature were included, and to try to exclude cases of interstitial pneumonia that occur most commonly in foals <1-mo-old (e.g., neonatal septicemias, congenital hyaline membrane disease).^10^

Within this age range, most of our cases were 2–4-mo-old Thoroughbred and Quarter Horse foals. This age distribution is similar to that reported in the literature,^10,15,26^ and the predominant breeds are representative of most of the horse submissions at CAHFS. Likewise, the geographic distribution of the cases matches the CAHFS laboratory that receives most equine submissions (the San Bernardino laboratory in southern California). Most of the foals were received in summer and spring, which is in agreement with the seasonal distribution of these cases in warmer months in California described previously.^15^ Hence, a contributory role of high environmental temperatures through heat-associated alveolar damage was suggested^15^; however, this seasonal distribution might just be representative of the foaling season, with a higher input of foals within the affected age range during spring and summer months.

The most commonly reported clinical signs among our foals were dyspnea/tachypnea and fever of ~40°C (104°F), and the clinical course of ~3.5 d, which is in agreement with previous reports.^13,15^ Antibiotic use was reported in approximately half of our cases, and the most commonly used antibiotics were penicillin and gentamicin, which are recommended for treating bronchopneumonia in foals.^29^ Erythromycin was suggested to play a role in some cases of interstitial/bronchointerstitial pneumonia of foals, potentially through suppressing neutrophil chemoattraction and phagocytosis,^15,18^ but its use was reported in only 3 of our cases. Similarly, corticosteroids are part of the treatment protocol of these cases,^13^ and corticosteroid use was reported in one-fourth of our foals. Because data on antibiotic and corticosteroid use were taken from the autopsy submission forms, it is possible that more cases were treated with these or other drugs, and it was not mentioned by the submitter.

Grossly, most lungs had a diffuse rubbery-to-firm texture, and more than half appeared non-collapsed. These are common gross features of interstitial pneumonia in animals^9^ and are consistent with the histologic features observed in our cases. In fact, we observed combinations of hyaline membranes, type II pneumocyte hyperplasia, and fibroplasia, which likely represent a continuum across the different phases of interstitial lung disease (i.e., exudative, proliferative, and fibrotic),^9^ and coincide with previous reports of this condition in foals.^15^ The interstitial component was not classifiable into any of the 3 phases in 2 cases. One of these cases had enterocolitis, but no other significant lesions were identified in the other case, and the cause of death was not determined. These cases might be consistent with foals in “recovery phase,” as reported in a 2021 article,^26^ and perhaps they died due to causes other than pneumonia.

Microscopic changes in bronchioles were observed in more than half of our cases, but mostly as peribronchiolar inflammatory infiltrates. Necrosis of the epithelium was rare, but hyperplasia was more common, which suggests that overall, the bronchiolar component was subacute-to-chronic, and perhaps the inciting etiologic agent was not present at the moment of examination and testing.^10^ Necrotizing bronchiolitis is a common feature described in some reports of bronchointerstitial pneumonias of unknown etiology in foals.^15,25^ In other reports, authors mentioned a predominantly interstitial pneumonia,^13,26^ which is more similar to most of our cases. Postmortem decomposition artifact and sampling area may prevent observation of subtle acute necrosis in the bronchiolar epithelium, and therefore, this feature might be more common than reported in our study.

More than half of our cases also had microscopic evidence of suppurative bronchopneumonia consistent with bacterial infection,^10^ which has been described in foals with interstitial or bronchointerstitial pneumonia.^13,26^ Likewise, 12% of our foals had pyogranulomas typical of *R. equi* infection. The extension of those lesions was probably not enough to justify a diffuse interstitial pneumonia per se; however, it remains possible that localized bacterial bronchopneumonia or *R. equi*–associated lesions incited a local ALI-ARDS–type response in some of the foals.^15^ In fact, bacteria are usually recovered from the lungs or transtracheal lavages in these cases,^13,15,26^ which coincides with our retrospective study, in which more than half of the foals had one or more bacteria isolated, predominantly *R. equi* and *E. coli*.

Some of our cases had concomitant conditions that may, in part or in whole, cause or contribute to interstitial pneumonia. *Pneumocystis* sp. forms, which can cause interstitial pneumonia in young foals and have been reported in studies similar to ours,^1,26^ were observed in 8 foals; however, in only one foal were *Pneumocystis* sp. forms generalized. In that case, the interstitial component was classified histologically as proliferative and fibrotic, which coincides with descriptions of *Pneumocystis* sp. pneumonia.^1,31^ There was one case of renal leptospirosis in our series. Pulmonary changes associated with *Leptospira* spp. in foals can range from alveolar hemorrhages to necrotizing interstitial pneumonia,^7^ and the case included in our study was classified as exudative and proliferative. Similarly, in 8 cases, the same bacteria were isolated from the lung and other parenchymal organ(s), which can be interpreted as bacteremia or septicemia. The latter can cause an ALI-ARDS reaction with morphologic features similar to those observed in the foals of our report.^10^

Even though a viral etiology for some of the cases cannot be totally excluded, no unequivocally causative viruses were identified, neither through the regular diagnostic workup, nor after testing a subset of the cases via herpesviral consensus PCR and deep sequencing and metagenomics. Known viruses associated with interstitial pneumonia in foals (i.e., EqAHV1, EqAHV4, equine IAV) were not detected; however, we understand the limitation that only a subset of cases was tested for these viruses during the regular diagnostic workup. In addition, there are other viruses that may contribute to respiratory disease, such as equine arteritis virus,^11^ equine rhinitis viruses,^12^ or equine adenoviruses,^10^ which were not included routinely in the respiratory panels of our laboratory system.

We performed our deep sequencing and metagenomics study on FFPE lung tissues, in which degradation may have prevented the detection of some viral sequences. Nevertheless, viral metagenomics from FFPE has been performed successfully with human tissues.^14^ In any case, our previous viral metagenomics study was more successful in identifying viruses using fresh tissues of foals with interstitial pneumonia,^2^ including 2 of the animals in our current metagenomics study.

EqGHV2 was detected in the samples of the 5 foals tested by herpesviral consensus PCR, but no viral nucleic acids were observed using ISH, and therefore this virus was considered unlikely to have contributed to the lesions. EqGHV2 is a very prevalent virus that may be detected by PCR in most foals from a certain age.^19^ Nevertheless, EqGHV2 qPCR demonstrated low Ct values in foals with features of viral interstitial pneumonia in a 2021 study,^26^ and a synergistic role between EqGHV2 and *R. equi* has been proposed elsewhere.^20^ Further studies using ISH and other methods of in situ detection in a larger cohort are warranted to definitively unravel the role of this virus in foals with interstitial or *R. equi*–associated pneumonia.

## Supplemental Material

sj-docx-2-vdi-10.1177_10406387251410524 – Supplemental material for Interstitial pneumonias of undetermined etiology in foals in California, 1990–2020 Supplemental material, sj-docx-2-vdi-10.1177_10406387251410524 for Interstitial pneumonias of undetermined etiology in foals in California, 1990–2020 by Javier Asin, Francisco Carvallo, Omar A. Gonzales-Viera, Melissa Macías-Rioseco, Nicolas Streitenberger, Sahar Abdelrazek, Beate Crossley, Patricia A. Pesavento and Francisco A. Uzal in Journal of Veterinary Diagnostic Investigation

sj-docx-3-vdi-10.1177_10406387251410524 – Supplemental material for Interstitial pneumonias of undetermined etiology in foals in California, 1990–2020 Supplemental material, sj-docx-3-vdi-10.1177_10406387251410524 for Interstitial pneumonias of undetermined etiology in foals in California, 1990–2020 by Javier Asin, Francisco Carvallo, Omar A. Gonzales-Viera, Melissa Macías-Rioseco, Nicolas Streitenberger, Sahar Abdelrazek, Beate Crossley, Patricia A. Pesavento and Francisco A. Uzal in Journal of Veterinary Diagnostic Investigation

sj-pdf-1-vdi-10.1177_10406387251410524 – Supplemental material for Interstitial pneumonias of undetermined etiology in foals in California, 1990–2020 Supplemental material, sj-pdf-1-vdi-10.1177_10406387251410524 for Interstitial pneumonias of undetermined etiology in foals in California, 1990–2020 by Javier Asin, Francisco Carvallo, Omar A. Gonzales-Viera, Melissa Macías-Rioseco, Nicolas Streitenberger, Sahar Abdelrazek, Beate Crossley, Patricia A. Pesavento and Francisco A. Uzal in Journal of Veterinary Diagnostic Investigation

## References

[1] Ainsworth DM , et al Recognition of *Pneumocystis carinii* in foals with respiratory distress. Equine Vet J 1993;25:103–108. 8467767 10.1111/j.2042-3306.1993.tb02917.x

[2] Altan E , et al New parvoviruses and picornavirus in tissues and feces of foals with interstitial pneumonia. Viruses 2021;13:1612. 34452477 10.3390/v13081612 PMC8402702

[3] Ames TR , et al Isolation of equine herpesvirus type 2 from foals with respiratory disease. Compend Contin Educ Pract Vet 1986;8:664–670.

[4] Barr BS. Pneumonia in weanlings. Vet Clin North Am Equine Pract 2003;19:35–49. 12747660 10.1016/s0749-0739(02)00065-2

[5] Breeze RG , et al Perilla ketone toxicity: a chemical model for the study of equine restrictive lung disease. Equine Vet J 1984;16:180–184. 6734583 10.1111/j.2042-3306.1984.tb01897.x

[6] Britton AP Robinson JH. Isolation of influenza A virus from a 7-day-old foal with bronchointerstitial pneumonia. Can Vet J 2002;43:55–56. 11802673 PMC339097

[7] Broux B , et al Acute respiratory failure caused by *Leptospira* spp. in 5 foals. J Vet Intern Med 2012;26:684–687. 22404543 10.1111/j.1939-1676.2012.00902.x

[8] Buergelt CD , et al A retrospective study of proliferative interstitial lung disease of horses in Florida. Vet Pathol 1986;23:750–756. 3811140 10.1177/030098588602300614

[9] Carvallo FR Stevenson VB. Interstitial pneumonia and diffuse alveolar damage in domestic animals. Vet Pathol 2022;59:586–601. 35253541 10.1177/03009858221082228

[10] Caswell JL Williams KJ. Respiratory system. In: Maxie MG ed. Jubb, Kennedy, & Palmer’s Pathology of Domestic Animals. Vol. 2. 6th ed. Elsevier, 2016:465–591.e464.

[11] Del Piero F . Equine viral arteritis. Vet Pathol 2000;37:287–296. 10896389 10.1354/vp.37-4-287

[12] Diaz-Méndez A , et al Characteristics of respiratory tract disease in horses inoculated with equine rhinitis A virus. Am J Vet Res 2014;75:169–178. 24471753 10.2460/ajvr.75.2.169

[13] Dunkel B , et al Acute lung injury/acute respiratory distress syndrome in 15 foals. Equine Vet J 2005;37:435–440. 16163946 10.2746/042516405774480094

[14] Gorißen C , et al Targeted whole-viral genome sequencing from formalin-fixed paraffin-embedded neuropathology specimens. Acta Neuropathol 2024;148:51. 39382575 10.1007/s00401-024-02812-z PMC11464609

[15] Lakritz J , et al Bronchointerstitial pneumonia and respiratory distress in young horses: clinical, clinicopathologic, radiographic, and pathological findings in 23 cases (1984–1989). J Vet Intern Med 1993;7:277–288. 8263846 10.1111/j.1939-1676.1993.tb01020.x

[16] Li H. Minimap2: pairwise alignment for nucleotide sequences. Bioinformatics 2018;34:3094–3100. 29750242 10.1093/bioinformatics/bty191 PMC6137996

[17] Murray MJ , et al Equine herpesvirus type 2: prevalence and seroepidemiology in foals. Equine Vet J 1996;28:432–436. 9049491 10.1111/j.2042-3306.1996.tb01614.x

[18] Nelson S , et al Erythromycin-induced suppression of pulmonary antibacterial defenses. A potential mechanism of superinfection in the lung. Am Rev Respir Dis 1987;136:1207–1212. 3314615 10.1164/ajrccm/136.5.1207

[19] Nordengrahn A , et al Prevalence of equine herpesvirus types 2 and 5 in horse populations by using type-specific PCR assays. Vet Res 2002;33:251–259. 12056476 10.1051/vetres:2002013

[20] Nordengrahn A , et al Equine herpesvirus type 2 (EHV-2) as a predisposing factor for *Rhodococcus equi* pneumonia in foals: prevention of the bifactorial disease with EHV-2 immunostimulating complexes. Vet Microbiol 1996;51:55–68. 8828122 10.1016/0378-1135(96)00032-6

[21] Patterson-Kane JC , et al The pathology of bronchointerstitial pneumonia in young foals associated with the first outbreak of equine influenza in Australia. Equine Vet J 2008;40:199–203. 18321807 10.2746/042516408X292214

[22] Pavulraj S , et al Equine herpesvirus type 4 (EHV-4) outbreak in Germany: virological, serological, and molecular investigations. Pathogens 2021;10:810. 34202127 10.3390/pathogens10070810 PMC8308676

[23] Perez-Ecija A , et al Equid herpesvirus 1 and *Rhodococcus equi* coinfection in a foal with bronchointerstitial pneumonia. J Vet Med Sci 2016;78:1511–1513. 27264610 10.1292/jvms.16-0024 PMC5059381

[24] Pesavento PA , et al In situ hybridization for localization of ovine herpesvirus 2, the agent of sheep-associated malignant catarrhal fever, in formalin-fixed tissues. Vet Pathol 2018;56:78–86. 30222071 10.1177/0300985818798085

[25] Prescott JF , et al Sporadic, severe bronchointerstitial pneumonia of foals. Can Vet J 1991;32:421–425. 17423819 PMC1480993

[26] Punsmann S , et al Acute interstitial pneumonia in foals: a severe, multifactorial syndrome with lung tissue recovery in surviving foals. Equine Vet J 2021;53:718–726. 32986272 10.1111/evj.13355

[27] Pusterla N , et al Investigation of three newly identified equine parvoviruses in blood and nasal fluid samples of clinically healthy horses and horses with acute onset of respiratory disease. Animals (Basel) 2021;11:3006. 34680025 10.3390/ani11103006 PMC8532786

[28] Rahman A , et al Retrospective study of pneumonia in non-racing horses in California. J Vet Diagn Invest 2022;34:587–593. 35535386 10.1177/10406387221094273 PMC9266512

[29] Reuss SM , et al Update on bacterial pneumonia in the foal and weanling. Vet Clin North Am Equine Pract 2015;31:121–135. 25600452 10.1016/j.cveq.2014.11.004

[30] Shapter FM , et al Equine Crofton weed (*Ageratina* spp.) pneumotoxicity: what do we know and what do we need to know? Animals (Basel) 2023;13:2082. 10.3390/ani13132082 PMC10339876 37443880

[31] Ueno T , et al *Pneumocystis* pneumonia in a Thoroughbred racehorse. J Equine Vet Sci 2014;34:333–336.

[32] VanDevanter DR , et al Detection and analysis of diverse herpesviral species by consensus primer PCR. J Clin Microbiol 1996;34:1666–1671. 8784566 10.1128/jcm.34.7.1666-1671.1996 PMC229091

[33] Wood DE , et al Improved metagenomic analysis with Kraken 2. Genome Biol 2019;20:257. 31779668 10.1186/s13059-019-1891-0 PMC6883579

