# Supplementary material for: Interstitial pneumonias of undetermined etiology in foals in California, 1990–2020
Source: J Vet Diagn Invest. 2026 Jan 29:10406387251410524. Online ahead of print. doi: 10.1177/10406387251410524 (PMC12858380; doi:10.1177/10406387251410524)
Supplement: sj-docx-2-vdi-10.1177_10406387251410524 – Supplemental material for Interstitial pneumonias of undetermined etiology in foals in California, 1990–2020 [file sj-docx-2-vdi-10.1177_10406387251410524.docx]

**Asin J, et al. Interstitial pneumonias of undetermined etiology in foals in California, 1990-2020**

**Supplemental material S1.** Methodology of deep sequencing and metagenomics study from formalin-fixed, paraffin embedded lung tissues

**Sample processing**

Scrolls were prepared and, after xylene deparaffinization, DNA was extracted using the QIAamp DNA FFPE Tissue Kit (Qiagen, Redwood City, CA, USA) and the concentration was quantified with a Qubit fluorometer (Thermo Fisher Scientific, Wilmington, DE, USA; Table 1).

**Nanopore sequencing**

The extracted DNA was then repaired using FFPE repair mix and purified using AMPure XP beads (Beckman Coulter Life Sciences, Indianapolis, IN, USA). For the PromethION run, samples were barcoded using the Native Barcoding Kit (SQK-NBD112.24). Barcoded samples were combined in a microcentrifuge tube and subjected to a second purification step using AMPure XP beads. Sequence adapters were then ligated to the pooled barcoded samples through mixing. Subsequently, the samples underwent a third purification step using AMPure XP beads and were finally eluted in 30 μL of elution buffer. For the MinION Flongle run, the sample was ligated to sequencing adapters using the SQK-LSK114 kit following FFPE DNA repair. The sample was then purified using AMPure XP beads, without barcoding, and eluted in 15 μL of elution buffer. Both MinION (FLO-MIN114) and PromethION (FLO-PRO114) R10.4.1 flow cells were utilized in this study. These flow cells were primed using the Priming Kit (ONT Ltd., EXP-FLP002) following standard ONT protocols. Before loading, the concentration and quality of the sequencing libraries were assessed using Qubit and TapeStation, then adjusted to meet Oxford Nanopore Technologies (ONT) platform requirements.

**Metagenomic read processing and pathogen identification**

FAST5 files obtained from MinION were base-called using ONT Guppy GPU (version 5.0.7) with the high-accuracy parameter.^5^ FASTQ files generated by PromethION were directly employed for analysis, as the PromethION's MinKNOW software default settings already provide high-accuracy base-calling.^4^ For pathogen identification, initially the FASTQ files were utilized using two different approaches.

In a first approach, we used the FASTQ files as an input for Kraken2 (v2.1.2)^6^ to assign taxonomy to sequencing reads and determine the taxonomic composition of the microbiomes within our samples utilizing the PlusPF Kraken2 (Refseq archaea, bacteria, viral, plasmid, human1, UniVec_Core, protozoa & fungi genome collection) and Viral (RefSeq Viral) databases as the references libraries. Subsequently, the output Kraken2 report files were uploaded to the Pavian Shiny App,^1^ which compiles the read counts for each species and allows for the visualization and comparison of calculated z-scores between read counts. In a second approach, Minimap2 (v2.24)^2^ was employed to map sequencing reads in FASTQ format against EHV5 (GCF_000929435.1) and EHV2 (GCF_000843985.2) genome downloaded from NCBI. Subsequently, Samtools (v1.16.1)^3^ was used to extract the mapped reads if present.

**Table 1.** DNA concentrations and sequencing platforms used

| **Case** | **Case type** | **Year of collection** | **DNA Extraction date** | **Qubit_DNA Concentration (ng/μL)** | **Sequencing_platform** | **Flow Cell cat No.** |
| --- | --- | --- | --- | --- | --- | --- |
| C1 | Control | 2020 | 05/24/24 | 18.2 | MinION | FLO-MIN114 |
| C2 | Control | 2018 | 05/24/24 | 5.02 | PromethION | FLO-PRO114M |
| 41 | Interstitial pneumonia | 2020 | 05/24/24 | 8.04 | PromethION | FLO-PRO114M |
| 39 | Interstitial pneumonia | 2020 | 05/24/24 | 45.2 | PromethION | FLO-PRO114M |
| 34 | Interstitial pneumonia | 2017 | 05/24/24 | 66.8 | PromethION | FLO-PRO114M |

**References**

1. Breitwieser FP, Salzberg SL. Pavian: interactive analysis of metagenomics data for microbiome studies and pathogen identification. *Bioinformatics*. 2020;36:1303-1304.

2. Li H. Minimap2: pairwise alignment for nucleotide sequences. *Bioinformatics*. 2018;34:3094-3100.

3. Li H, et al. The Sequence Alignment/Map format and SAMtools. *Bioinformatics*. 2009;25:2078-9.

4. Loose M, et al. Real-time selective sequencing using nanopore technology. *Nat Methods*. 2016;13:751-4.

5. Wick RR, et al. Completing bacterial genome assemblies with multiplex MinION sequencing. *Microb Genom*. 2017;3:e000132.

6. Wood DE, et al. Improved metagenomic analysis with Kraken 2. *Genome Biol*. 2019;20:257.
