# Supplementary material for: Interstitial pneumonias of undetermined etiology in foals in California, 1990–2020
Source: J Vet Diagn Invest. 2026 Jan 29:10406387251410524. Online ahead of print. doi: 10.1177/10406387251410524 (PMC12858380; doi:10.1177/10406387251410524)
Supplement: sj-docx-3-vdi-10.1177_10406387251410524 – Supplemental material for Interstitial pneumonias of undetermined etiology in foals in California, 1990–2020 [file sj-docx-3-vdi-10.1177_10406387251410524.docx]

| **Case**  **Asin J, et al. Interstitial pneumonias of undetermined etiology in foals in California, 1990-2020**  Supplemental Table 1. Main pulmonary microscopic lesions, findings in other organs, and ancillary test results in foals with interstitial/bronchointerstitial pneumonia | **Interstitial pneumonia phase** | **Suppurative bronchopneumonia** | **Pulmonary abscess/pyogranuloma** | **Lesions in extrathoracic organs** | **Culture results in lung** | **Culture results in extrapulmonary sites** | ***Salmonella* sp screen/culture** | **EHV-1 FA/IHC/PCR** | **EHV-4 IHC/PCR** | **Influenza A PCR** | **Virus isolation** | **Selenium deficiency** | **Other hepatic mineral or vitamin deficiencies** |
| --- | --- | --- | --- | --- | --- | --- | --- | --- | --- | --- | --- | --- | --- |
| **1** | E+P | NO | NO | Not available | No growth | Not available | ND | ND | ND | ND | ND | ND | ND |
| **2** | E+P | YES | NO | None | *Streptococcus zooepidemicus* (Lg#), *Actinobacillus* sp. (Sm#), *Staphylococcus aureus* (Sm#) | Liver: *Actinobacillus* spp. (Sm#), *Streptococcus zooepidemicus* (Sm#) Lymph node: *Actinobacillus sp.* (Rare), *Staphylococcus aureus* (Rare), *Streptococcus zooepidemicus* (Rare) | Negative (Tissue pool, gut pool) | ND | ND | ND | Negative | ND | ND |
| **3** | E | YES | NO | Guttural pouch, spleen, colon: Lymphoid hyperplasia | No growth | Liver, spleen, lymph node (bronchial): No growth | Negative (Liver, small intestine) | ND | ND | ND | Negative | ND | ND |
| **4** | E+P | YES | NO | Bronchial lymph nodes: Hemorrhage | *Rhodococcus equi* (Sm#) | Liver: No growth Lymph node: Mixed flora, *Streptococcus zooepidemicus*, *Actinobacillus* sp. | Negative (Tissue pool) | ND | ND | ND | ND | ND | ND |
| **5** | E+P | YES | NO | None | Mixed flora (rare) | Liver, spleen: No growth | Negative (Tissue pool, gut pool) | ND | ND | ND | Negative | ND | ND |
| **6** | NC | NO | NO | Small intestine: Coccidia, mild numbers (*Eimeria leukharti*) Heart, liver: mild, multifocal mixed myocarditis and hepatitis | No growth | Liver, kidney, lymph node (mesenteric): No growth | Negative (Tissue pool, gut pool) | Not detected | ND | ND | Negative | ND | ND |
| **7** | E+P+F | NO | NO | Liver: Centrilobular degeneration and necrosis | *Rhodococcus equi* (rare) | Liver: No growth | ND | ND | ND | ND | ND | ND | ND |
| **8** | E+P+F | YES | NO | Not available | No growth | Not done | ND | Not detected | Not detected | ND | ND | ND | ND |
| **9** | E+P+F | NO | YES | Spleen, colon (MALT): Lymphoid depletion | *Rhodococcus equi*, mixed flora | Liver: Mixed flora (rare) | Negative (Gut pool, lung) | Not detected | ND | ND | ND | ND | ND |
| **10** | E+P | YES | NO | Kidney: Tubular degeneration and necrosis | No growth | Pericardium: No growth | ND | ND | ND | ND | ND | ND | ND |
| **11** | E+P+F | NO | NO | None | No growth | Liver, spleen, brain: No growth Kidney: *Escherichia coli* | Negative (Liver) | ND | ND | ND | Negative | Yes | No |
| **12** | E+P | YES | NO | None | *E. coli* (rare), mixed flora (rare) | Not done | ND | Not detected | ND | ND | ND | ND | ND |
| **13** | E+P+F | YES | NO | Kidney: Proteinaceous tubular casts | *E. coli* (Sm#), mixed flora (rare) | Liver: *E. coli* (small numbers), mixed flora (rare) | Negative (Colon) | Not detected | ND | ND | ND | ND | ND |
| **14** | E+P+F | YES | NO | Heart: Focal, mild endocarditis  Spleen, colon (MALT), thymus: Lymphoid depletion | *Bordetella bronchiseptica* (Sm# to Mod#), mixed flora (rare) | Liver: Mixed flora (rare) Spleen, lymph nodes (thoracic, bronchial): *Bordetella bronchiseptica* (rare to Sm#, mixed flora (rare) | Negative (Feces) | Not detected | ND | Not detected | Negative | ND | ND |
| **15** | E+P+F | YES | NO | Spleen, colon (MALT), lymph nodes, thymus: Lymphoid depletion | *Bordetella bronchiseptica* (Sm#) | Liver: *Bordetella bronchiseptica* (rare), mixed flora (rare) Lymph node (retropharyngeal): No growth | ND | Not detected | Not detected | Not detected | ND | ND | ND |
| **16** | E+P | YES | NO | Spleen, lymph nodes: Lymphoid depletion | Mixed flora (rare) | Liver: Mixed flora (rare) Lymph node: Mixed flora (rare), *Streptococcus zooepidemicus* (rare) | ND | Not detected | Not detected | Not detected | ND | ND | ND |
| **17** | E+P+F | YES | NO | None | No growth | Colon: Mixed flora (Sm#) | Negative (Colon) | Not detected | Not detected | Not detected | ND | Yes | Low vitamin E (1.7 ppm) |
| **18** | P+F | NO | NO | None | *Enterococcus* sp. (Lg#) | Liver: Mixed flora (rare) | Negative (Liver, colon) | ND | ND | ND | Negative | ND | ND |
| **19** | P | NO | NO | None | *Streptococcus zooepidemicus* (Sm#), *Actinobacillus* sp. (Sm#),  mixed flora | Liver: Mixed flora (Sm#), *Streptococcus zooepidemicus* (Sm#) | Negative (Liver, colon) | ND | ND | ND | ND | ND | ND |
| **20** | P+F | YES | NO | Liver: Necrosis,multifocal rare with neutrophilic infiltrates Spleen, lymph nodes: Lymphoid hyperplasia  Stomach: Ulcer, focal | No growth | Liver: No growth Cecum: Mixed flora (rare) | Negative (Liver, lymph node, cecum) | Not detected | Not detected | ND | Negative | ND | ND |
| **21** | E+P | YES | YES | Kidney: Infarcts, multifocal, acute Liver: Centrilobular degeneration and necrosis | *Rhodococcus equi* (Md#) | Liver: No growth Cecum: Mixed flora (rare) | Negative (Liver, lymph node, cecum) | Not detected | ND | ND | ND | ND | ND |
| **22** | E | YES | NO | Lymph nodes: Lymphoid depletion | *E. coli* (Sm# to Md#) | Not done | Negative (Lung) | Not detected | ND | ND | Negative | Yes | No |
| **23** | E+P | NO | YES | Small intestine: Intussusception | *Rhodococcus equi* [Pyogranuloma] (Lg#), *Klebsiella* sp. [lung tissue and pyogranuloma] (Md#) | Lymph node (bronchial): *Rhodococcus equi* | Negative (feces) | Not detected | ND | ND | ND | Yes | No |
| **24** | NC | NO | NO | Spleen: Lymphoid depletion Colon: Colitis, ulcerative, focally extensive  Small intestine: Suppurative cryptititis with coccidia Kidney: Tubular degeneration and necrosis with crystals; renal infarcts | No growth | Liver, lung: No growth | Negative (Intestinal contents) | Not detected | Not detected | ND | ND | Yes | No |
| **25** | E+P+F | NO | NO | Liver: Hepatitis, suppurative, multifocal | *E. coli* (Md#), mixed flora (Md#) | Liver:  *E. coli* (Md #), mixed flora (small #), | Negative (Liver, colon) | ND | ND | ND | ND | ND | ND |
| **26** | E | NO | NO | 6th cervical vertebra: Osteomyelitis, suppurative with bacteria Liver: Necrosis, mutifocal | *Rhodococcus equi* (Md#), mixed flora (Sm#) | Liver: *Rhodococcus equi* (rare) Spine pyogranuloma: *Rhodococcus equi* (Lg#) | Negative (Small intestine) | Not detected | ND | ND | ND | Yes | No |
| **27** | E+P | NO | NO | Mesenteric lymph nodes: Lymphadenitis, necrosuppurative with bacteria | *E. coli* (Md#) | Colon pyogranuloma: *Rhodococcus equi* (Md#) | Negative (Colon) | ND | ND | ND | ND | ND | ND |
| **28** | P | NO | NO | Abdominal cavity: Peritonitis, fibrinous, mild | Mixed flora (rare) | Liver: Mixed flora (Sm#) Peritoneum: Mixed flora (rare), *Streptococcus* *zooepidemicus* (rare) Colon: No anaerobic bacteria or *C. difficile* detected | Negative (Liver) | Not detected | ND | Not detected | Negative | Yes | No |
| **29** | E+P | NO | NO | Stomach: Gastritis, neutrophilic, focal | No growth | Liver: Mixed flora (rare) Small intestine: Mixed flora rare (aerobic and anaerobic); No. *C. difficile* detected | Negative (Liver, small intestine) | Not detected | ND | ND | ND | ND | ND |
| **30** | E+P | YES | NO | Spleen: Lymphoid depletion | *Rhodococcus equi* (Sm#), *E. coli* (rare), mixed flora (rare) | Spleen: Mixed flora (rare), *E. coli*  Lymph node (bronchial): *Rhodococcus equi*, no anaerobic bacteria isolated Guttural puch: Mixed flora (Mod #), *E. coli* (Md#) | Negative (Colon, lung) | Not detected | ND | Not detected | Negative | Yes | No |
| **31** | E+P | YES | NO | None | No growth | Liver: No growth Kidney: Mixed flora (rare) | Negative (Liver) | Not detected | ND | Not detected | ND | ND | ND |
| **32** | E | NO | YES | Liver: Centrilobular degeneration and necrosis | No growth | Liver: No growth | ND | Not detected | ND | Not detected | Negative | ND | ND |
| **33** | E+P+F | YES | NO | Liver: Centrilobular degeneration and necrosis | No growth | Liver: No growth Lymph node (bronchial): *E. coli* Spleen: Mixed flora (rare) | Negative (feces) | Not detected | ND | Not detected | Negative | Yes | No |
| **34** | E+P+F | NO | NO | Liver: Centrilobular degeneration and necrosis  Stomach: Ulcers, multifocal | No growth | Liver: No growth Small intestine, cecum: Mixed flora (Lg#) | Negative (Small intestine) | Not detected | ND | Not detected | Negative | Yes | No |
| **35** | E+P | NO | YES | None | No growth | Liver: No growth | Negative (Colon) | Not detected | Not detected | Not detected | Negative | No | No |
| **36** | E+P | YES | NO | Kidney: Tubulointerstitial nephritis, *Leptospira* sp IHC positive  Small intestine: Roundworms (*Parascaris* sp), large numbers; lymphoid depletion (MALT) | No growth | Not done | Negative (Feces) | Not detected | ND | Not detected | ND | No | No |
| **37** | E+P+F | YES | NO | Liver: Necrosis, random, rare and fibrinocellular thrombi Small intestines: Roundworms, small numbers (*Parascaris* sp) | No growth | Not done | ND | Not detected | Not detected | Not detected | ND | Yes | No |
| **38** | P+F | NO | NO | Abdominal cavity: Intestinal perforation with fibrinous peritonitis Mesenteric lymph nodes: Lymphoid hyperplasia  Spleen: Lymphoid depletion | Mixed flora (rare) | Liver: *Enterococcus hirae* (Sm#), *E. coli* (Sm#) Small intestine, colon: Mixed flora (Lg#), *E. coli* (Sm#) Abdominal cavity: *Enterococcus hirae* (Lg#), *E. coli* (Lg#) Joint swab: *Staphylococcus haemolyticus* (rare#), Mixed flora (Sm#), *E. coli* (Sm#) | Negative (Liver, small intestine, colon) | Not detected | Not detected | Not detected | ND | No | No |
| **39** | E | YES | NO | Small intestine: Roundworms, large numbers (*Parascaris* sp) | No growth | Liver: No growth | Negative (Liver) | Not detected | Not detected | Not detected | Negative | ND | No |
| **40** | P+F | NO | NO | Liver: Necrosis, random, very rare | No growth | Liver: *E. coli* (Sm#) | Negative (Liver) | Not detected | Not detected | Not detected | ND | Yes | No |
| **41** | E+P+F | YES | NO | Liver: Centrilobular degeneration and necrosis; hepatic necrosis, very rare with neutrophils Spleen: Lymphoid depletion | *Acinetobacter Iwofii* (Sm#), mixed flora (rare) | Liver: *E. coli* (Sm#) | Negative (Small intestine) | Not detected | Not detected | Not detected | Negative | No | No |

E: Exudative; F: Fibrotic; Lg#: Large numbers; Md#: Moderate numbers; NC: Non-classifiable; ND: Not done; P: Proliferative; R: Reparative; Sm#: Small numbers
