# Supplementary material for: Interstitial pneumonias of undetermined etiology in foals in California, 1990–2020
Source: J Vet Diagn Invest. 2026 Jan 29:10406387251410524. Online ahead of print. doi: 10.1177/10406387251410524 (PMC12858380; doi:10.1177/10406387251410524)
Supplement: sj-pdf-1-vdi-10.1177_10406387251410524 – Supplemental material for Interstitial pneumonias of undetermined etiology in foals in California, 1990–2020 [file sj-pdf-1-vdi-10.1177_10406387251410524.pdf]

Asin J, et al. Interstitial pneumonias of undetermined etiology in foals in California, 1990–2020

Supplemental material. Methodology of deep sequencing and metagenomics study from formalin-fixed, paraffin-embedded (FFPE) lung tissues

Sample processing

Scrolls were prepared and, after xylene deparaffinization, DNA was extracted (QIAamp DNA FFPE tissue kit; Qiagen) and the concentration was quantified (Qubit fluorometer, ThermoFisher; Suppl. Table 1).

Nanopore sequencing

The extracted DNA was repaired (FFPE repair mix) and purified (AMPure XP beads; Beckman Coulter). For the PromethION run, samples were barcoded (Native barcoding kit, SQK-NBD112.24; Oxford Nanopore Technologies [ONT]). Barcoded samples were combined in a microcentrifuge tube and subjected to a second purification step using AMPure XP beads. Sequence adapters were then ligated to the pooled barcoded samples through mixing. Subsequently, the samples underwent a third purification step using AMPure XP beads and were finally eluted in 30 µL of elution buffer. For the MinION Flongle run, the sample was ligated to sequencing adapters using the SQK-LSK114 kit following FFPE DNA repair. The sample was then purified using AMPure XP beads, without barcoding, and eluted in 15 µL of elution buffer. Both MinION (FLO-MIN114) and PromethION (FLO-PRO114) R10.4.1 flow cells were utilized in our study. These flow cells were primed using the Priming kit (ONT, EXP-FLP002) following standard ONT protocols. Before loading, the concentration and quality of the sequencing

libraries were assessed using Qubit and TapeStation (Agilent) , then adjusted to meet ONT platform requirements.

Metagenomic read processing and pathogen identification

FAST5 files obtained from MinION were base-called using ONT Guppy GPU (v.5.0.7) with the high-accuracy parameter.[5] FASTQ files generated by PromethION were directly employed for analysis; the PromethION MinKNOW software default settings provide high-accuracy base-calling.[4] For pathogen identification, initially, the FASTQ files were utilized using 2 different approaches.

In a first approach, we used the FASTQ files as an input for Kraken2 (v.2.1.2)[6] to assign taxonomy to sequencing reads and determine the taxonomic composition of the microbiomes within our samples, utilizing the PlusPF Kraken2 (Refseq archaea, bacteria, viral, plasmid, human1, UniVec_Core, protozoa and fungi genome collection) and Viral (RefSeq Viral) databases as the references libraries. Subsequently, the output Kraken2 report files were uploaded to the Pavian Shiny App,[1] which compiles the read counts for each species and allows the visualization and comparison of calculated *z*-scores between read counts. In a second approach, Minimap2 (v.2.24)[2] was employed to map sequencing reads in FASTQ format against EqGHV5 (GCF_000929435.1) and EqGHV2 (GCF_000843985.2) genome downloaded from NCBI. Subsequently, Samtools (v.1.16.1)[3] was used to extract the mapped reads if present.

Supplemental Table 1. DNA concentrations and sequencing platforms used.

Case	Case type	Year of collection	DNA extraction date	Qubit_DNA concentration, ng/μL	Sequencing platform	Flow cell catalog no.
C1	Control	2020	2024 May 24	18.2	MinION	FLO-MIN114
C2	Control	2018	2024 May 24	5.02	PromethION	FLO-PRO114M
34	Interstitial pneumonia	2017	2024 May 24	66.8	PromethION	FLO-PRO114M
39	Interstitial pneumonia	2020	2024 May 24	45.2	PromethION	FLO-PRO114M
41	Interstitial pneumonia	2020	2024 May 24	8.04	PromethION	FLO-PRO114M

Supplemental Table 2. Main pulmonary microscopic lesions, findings in other organs, and ancillary test results in foals with interstitial/bronchointerstitial pneumonia

Case	Interstitial pneumonia phase	Suppurative bronchopneumonia	Pulmonary abscess/ pyogranuloma	Lesions in extrathoracic organs	Culture results in lung	Culture results in extrapulmonary sites	*Salmonella* sp. screen/ culture	EqAHV1 FA/IHC/PCR	EqAHV-4 IHC/PCR	IAV PCR	Virus isolation	Selenium deficiency	Other hepatic mineral or vitamin deficiencies
**1**	E+P	No	No	Not available	No growth	Not available	ND	ND	ND	ND	ND	ND	ND
**2**	E+P	Yes	No	None	*Streptococcus zooepidemicus* (Lg#), *Actinobacillus* sp. (Sm#), *Staphylococcus aureus* (Sm#)	Liver: *Actinobacillus* spp. (Sm#), *Streptococcus zooepidemicus* (Sm#) Lymph node: *Actinobacillus* sp. (Rare), *Staphylococcus aureus* (Rare), *Streptococcus zooepidemicus* (Rare)	– (tissue pool, gut pool)	ND	ND	ND	–	ND	ND
**3**	E	Yes	No	Guttural pouch, spleen, colon: lymphoid hyperplasia	No growth	Liver, spleen, lymph node (bronchial): no growth	– (liver, small intestine)	ND	ND	ND	–	ND	ND
**4**	E+P	Yes	No	Bronchial lymph nodes: hemorrhage	*Rhodococcus equi* (Sm#)	Liver: no growth Lymph node: mixed flora, *Streptococcus zooepidemicus*, *Actinobacillus* sp.	– (tissue pool)	ND	ND	ND	ND	ND	ND
**5**	E+P	Yes	No	None	Mixed flora (rare)	Liver, spleen: no growth	– (tissue pool, gut pool)	ND	ND	ND	–	ND	ND

**6**	NC	No	No	Small intestine: coccidia, mild numbers (*Eimeria leuckarti*)<br>Heart, liver: mild, multifocal mixed myocarditis and hepatitis	No growth	Liver, kidney, lymph node (mesenteric): no growth	– (tissue pool, gut pool)	Not detected	ND	ND	–	ND	ND
**7**	E+P+F	No	No	Liver: centrilobular degeneration and necrosis	*Rhodococcus equi* (rare)	Liver: no growth	ND	ND	ND	ND	ND	ND	ND
**8**	E+P+F	Yes	No	Not available	No growth	Not done	ND	Not detected	Not detected	ND	ND	ND	ND
**9**	E+P+F	No	Yes	Spleen, colon (MALT): lymphoid depletion	*Rhodococcus equi*, mixed flora	Liver: mixed flora (rare)	– (gut pool, lung)	Not detected	ND	ND	ND	ND	ND
**10**	E+P	Yes	No	Kidney: tubular degeneration and necrosis	No growth	Pericardium: no growth	ND	ND	ND	ND	ND	ND	ND
**11**	E+P+F	No	No	None	No growth	Liver, spleen, brain: no growth<br>Kidney: *Escherichia coli*	– (liver)	ND	ND	ND	–	Yes	No
**12**	E+P	Yes	No	None	*E. coli* (rare), mixed flora (rare)	Not done	ND	Not detected	ND	ND	ND	ND	ND
**13**	E+P+F	Yes	No	Kidney: proteinaceous tubular casts	*E. coli* (Sm#), mixed flora (rare)	Liver: *E. coli* (Sm#), mixed flora (rare)	– (colon)	Not detected	ND	ND	ND	ND	ND
**14**	E+P+F	Yes	No	Heart: focal, mild endocarditis<br>Spleen, colon (MALT), thymus: lymphoid depletion	*Bordetella bronchiseptica* (Sm# to Md#), mixed flora (rare)	Liver: mixed flora (rare)<br>Spleen, lymph nodes (thoracic, bronchial): *Bordetella bronchiseptica*	– (feces)	Not detected	ND	Not detected	–	ND	ND

						(rare to Sm#, mixed flora (rare)							
**15**	E+P+F	Yes	No	Spleen, colon (MALT), lymph nodes, thymus: lymphoid depletion	*Bordetella bronchiseptica* (Sm#)	Liver: *Bordetella bronchiseptica* (rare), mixed flora (rare)<br>Lymph node (retropharyngeal): no growth	ND	Not detected	Not detected	Not detected	ND	ND	ND
**16**	E+P	Yes	No	Spleen, lymph nodes: lymphoid depletion	Mixed flora (rare)	Liver: mixed flora (rare)<br>Lymph node: mixed flora (rare), *Streptococcus zooepidemicus* (rare)	ND	Not detected	Not detected	Not detected	ND	ND	ND
**17**	E+P+F	Yes	No	None	No growth	Colon: mixed flora (Sm#)	– (colon)	Not detected	Not detected	Not detected	ND	Yes	Low vitamin E (1.7 ppm)
**18**	P+F	No	No	None	*Enterococcus* sp. (Lg#)	Liver: mixed flora (rare)	– (liver, colon)	ND	ND	ND	–	ND	ND
**19**	P	No	No	None	*Streptococcus zooepidemicus* (Sm#), *Actinobacillus* sp. (Sm#), mixed flora	Liver: mixed flora (Sm#), *Streptococcus zooepidemicus* (Sm#)	– (liver, colon)	ND	ND	ND	ND	ND	ND
**20**	P+F	Yes	No	Liver: necrosis, random, rare with neutrophilic infiltrates<br>Spleen, lymph nodes: lymphoid hyperplasia<br>Stomach: ulcer, focal	No growth	Liver: no growth<br>Cecum: mixed flora (rare)	– (liver, lymph node, cecum)	Not detected	Not detected	ND	–	ND	ND

**21**	E+P	Yes	Yes	Kidney: infarcts, multifocal, acute Liver: centrilobular degeneration and necrosis	*Rhodococcus equi* (Md#)	Liver: no growth Cecum: mixed flora (rare)	– (liver, lymph node, cecum)	Not detected	ND	ND	ND	ND	ND
**22**	E	Yes	No	Lymph nodes: lymphoid depletion	*E. coli* (Sm# to Md#)	Not done	– (lung)	Not detected	ND	ND	–	Yes	No
**23**	E+P	No	Yes	Small intestine: intussusception	*Rhodococcus equi* [pyogranuloma] (Lg#), *Klebsiella* sp. [lung tissue and pyogranuloma] (Md#)	Lymph node (bronchial): *Rhodococcus equi*	– (feces)	Not detected	ND	ND	ND	Yes	No
**24**	NC	No	No	Spleen: lymphoid depletion Colon: colitis, ulcerative, focally extensive Small intestine: suppurative cryptitis with coccidia Kidney: tubular degeneration and necrosis with crystals; renal infarcts	No growth	Liver, lung: no growth	– (intestinal contents)	Not detected	Not detected	ND	ND	Yes	No
**25**	E+P+F	No	No	Liver: hepatitis, suppurative, random	*E. coli* (Md#), mixed flora (Md#)	Liver: *E. coli* (Md#), mixed flora (Sm#),	– (liver, colon)	ND	ND	ND	ND	ND	ND
**26**	E	No	No	6th cervical vertebra: osteomyelitis, suppurative with bacteria Liver: necrosis, random	*Rhodococcus equi* (Md#), mixed flora (Sm#)	Liver: *Rhodococcus equi* (rare) Spine pyogranuloma: *Rhodococcus equi* (Lg#)	– (small intestine)	Not detected	ND	ND	ND	Yes	No

**27**	E+P	No	No	Mesenteric lymph nodes: lymphadenitis, necrosuppurative with bacteria	*E. coli* (Md#)	Colon pyogranuloma: *Rhodococcus equi* (Md#)	– (colon)	ND	ND	ND	ND	ND	ND
**28**	P	No	No	Abdominal cavity: peritonitis, fibrinous, mild	Mixed flora (rare)	Liver: mixed flora (Sm#) Peritoneum: mixed flora (rare), *Streptococcus zooepidemicus* (rare) Colon: no anaerobic bacteria or *C. difficile* detected	– (liver)	Not detected	ND	Not detected	–	Yes	No
**29**	E+P	No	No	Stomach: gastritis, neutrophilic, focal	No growth	Liver: mixed flora (rare) Small intestine: mixed flora rare (aerobic and anaerobic); no *C. difficile* detected	– (liver, small intestine)	Not detected	ND	ND	ND	ND	ND
**30**	E+P	Yes	No	Spleen: lymphoid depletion	*Rhodococcus equi* (Sm#), *E. coli* (rare), mixed flora (rare)	Spleen: mixed flora (rare), *E. coli* Lymph node (bronchial): *Rhodococcus equi*, no anaerobic bacteria isolated Guttural pouch: mixed flora (Md#), *E. coli* (Md#)	– (colon, lung)	Not detected	ND	Not detected	–	Yes	No
**31**	E+P	Yes	No	None	No growth	Liver: no growth Kidney: mixed flora (rare)	– (liver)	Not detected	ND	Not detected	ND	ND	ND

**32**	E	No	Yes	Liver: centrilobular degeneration and necrosis	No growth	Liver: no growth	ND	Not detected	ND	Not detected	–	ND	ND
**33**	E+P+F	Yes	No	Liver: centrilobular degeneration and necrosis	No growth	Liver: no growth<br>Lymph node (bronchial): *E. coli*<br>Spleen: Mixed flora (rare)	– (feces)	Not detected	ND	Not detected	–	Yes	No
**34**	E+P+F	No	No	Liver: centrilobular degeneration and necrosis<br>Stomach: ulcers, multifocal	No growth	Liver: no growth<br>Small intestine, cecum: mixed flora (Lg#)	– (small intestine)	Not detected	ND	Not detected	–	Yes	No
**35**	E+P	No	Yes	None	No growth	Liver: no growth	– (colon)	Not detected	Not detected	Not detected	–	No	No
**36**	E+P	Yes	No	Kidney: tubulointerstitial nephritis, *Leptospira* sp. IHC positive<br>Small intestine: roundworms (*Parascaris* sp.; Lg#); lymphoid depletion (MALT)	No growth	Not done	– (feces)	Not detected	ND	Not detected	ND	No	No
**37**	E+P+F	Yes	No	Liver: necrosis, random, rare and fibrinocellular thrombi<br>Small intestines: roundworms (*Parascaris* sp.; Sm#)	No growth	Not done	ND	Not detected	Not detected	Not detected	ND	Yes	No

**38**	P+F	No	No	Abdominal cavity: intestinal perforation with fibrinous peritonitis Mesenteric lymph nodes: lymphoid hyperplasia Spleen: lymphoid depletion	Mixed flora (rare)	Liver: *Enterococcus hirae* (Sm#), *E. coli* (Sm#) Small intestine, colon: mixed flora (Lg#), *E. coli* (Sm#) Abdominal cavity: *Enterococcus hirae* (Lg#), *E. coli* (Lg#) Joint swab: *Staphylococcus haemolyticus* (rare#), mixed flora (Sm#), *E. coli* (Sm#)	– (liver, small intestine, colon)	Not detected	Not detected	Not detected	ND	No	No
**39**	E	Yes	No	Small intestine: roundworms (*Parascaris* sp.; Lg#)	No growth	Liver: no growth	– (liver)	Not detected	Not detected	Not detected	–	ND	No
**40**	P+F	No	No	Liver: necrosis, random, very rare	No growth	Liver: *E. coli* (Sm#)	– (liver)	Not detected	Not detected	Not detected	ND	Yes	No
**41**	E+P+F	Yes	No	Liver: centrilobular degeneration and necrosis; hepatic necrosis, very rare with neutrophils Spleen: lymphoid depletion	*Acinetobacter iwofii* (Sm#), mixed flora (rare)	Liver: *E. coli* (Sm#)	– (small intestine)	Not detected	Not detected	Not detected	–	No	No

E = exudative; F = fibrotic; Lg# = large numbers; Md# = moderate numbers; NC = non-classifiable; ND = not done; P = proliferative; R = reparative; Sm# = small numbers; – = negative.

References

1. Breitwieser FP, Salzberg SL. Pavian: interactive analysis of metagenomics data for microbiome studies and pathogen identification. Bioinformatics 2020;36:1303–1304.
2. Li H. Minimap2: pairwise alignment for nucleotide sequences. Bioinformatics 2018;34:3094–3100.
3. Li H, et al. The Sequence Alignment/Map format and SAMtools. Bioinformatics 2009;25:2078–2079.
4. Loose M, et al. Real-time selective sequencing using nanopore technology. Nat Methods 2016;13:751–754.
5. Wick RR, et al. Completing bacterial genome assemblies with multiplex MinION sequencing. Microb Genom 2017;3:e000132.
6. Wood DE, et al. Improved metagenomic analysis with Kraken 2. Genome Biol 2019;20:257.
